# Acute Tubular Injury is Associated With Severe Traumatic Brain Injury: *in Vitro* Study on Human Tubular Epithelial Cells

**DOI:** 10.1038/s41598-019-42147-4

**Published:** 2019-04-15

**Authors:** Federica Civiletti, Barbara Assenzio, Anna Teresa Mazzeo, Davide Medica, Fulvia Giaretta, Ilaria Deambrosis, Vito Fanelli, Vito Marco Ranieri, Vincenzo Cantaluppi, Luciana Mascia

**Affiliations:** 1 Department of Anesthesia and Intensive Care, University of Turin, Città della Salute e della Scienza – Molinette University Hospital, Turin, Italy; 2 Center for Experimental Medical Research (CeRMS), University of Turin, Città della Salute e della Scienza- Molinette University Hospital, Turin, Italy; 3 Nephrology, Dialysis and Kidney Transplantation Unit, Department of Translational Medicine, University of Eastern Piedmont “A. Avogadro”, Maggiore della Carità University Hospital, Novara, Italy; 4 Anesthesia and Intensive Care, Department of Medical and Surgical Science and Biotechnologies, University of Rome La Sapienza, Rome, Italy; 5 Anesthesia and Intensive Care, Departments of Anesthesiology, Critical Care, and Pain Medicine University of Rome La Sapienza, Rome, Italy

## Abstract

Acute kidney injury following traumatic brain injury is associated with poor outcome. We investigated *in vitro* the effects of plasma of brain injured patients with acute tubular kidney injury on kidney tubular epithelial cell function. we performed a prospective observational clinical study in ICU in a trauma centre of the University hospital in Italy including twenty-three ICU patients with traumatic brain injury consecutively enrolled. Demographic data were recorded on admission: age 39 ± 19, Glasgow Coma Score 5 (3–8). Neutrophil Gelatinase-Associated Lipocalin and inflammatory mediators were measured in plasma on admission and after 24, 48 and 72 hours; urine were collected for immunoelectrophoresis having healthy volunteers as controls. Human renal proximal tubular epithelial cells were stimulated with patients or controls plasma. Adhesion of freshly isolated human neutrophils and trans-epithelial electrical resistance were assessed; cell viability (XTT assay), apoptosis (TUNEL staining), Neutrophil Gelatinase-Associated Lipocalin and Megalin expression (quantitative real-time PCR) were measured. All patients with normal serum creatinine showed increased plasmatic Neutrophil Gelatinase-Associated Lipocalin and increased urinary Retinol Binding Protein and α1-microglobulin. Neutrophil Gelatinase-Associated Lipocalin was significantly correlated with both inflammatory mediators and markers of tubular damage. Patient’ plasma incubated with tubular cells significantly increased adhesion of neutrophils, reduced trans-epithelial electrical resistance, exerted a cytotoxic effect and triggered apoptosis and down-regulated the endocytic receptor Megalin compared to control. Plasma of brain injured patients with increased markers of subclinical acute kidney induced a pro-inflammatory phenotype, cellular dysfunction and apoptotic death in tubular epithelial cells.

## Introduction

In patients with traumatic brain injury (TBI), outcome is highly influenced by non-neurological complications^1–3^ including acute kidney injury (AKI)^4^. Recent studies reported an incidence of AKI equal to 8–20% in TBI patients^5,6^ using serum creatinine increase and urine output decrease as criteria for AKI. However it has been reported that 15–20% of patients who do not fulfill these criteria are likely to have acute tubular damage, a condition recently defined as “subclinical AKI”^7^.

In severe brain injured patients, a systemic inflammatory reaction, triggered by the primary injury and associated with secondary insults^8^, is mediated by the release of both pro- and anti- inflammatory cytokines and chemokines^9–12^. Interleukin-6 (IL-6), up-regulated in human serum and cerebrospinal fluid following TBI^13–15^, is also expressed in renal tubular epithelial cells during AKI, promoting neutrophil infiltration and exacerbating renal injury^16^. Consequently, systemic inflammation occurring after TBI has been proposed as a possible mechanism of AKI causing functional alterations and apoptosis of tubular epithelial cells similarly to the sepsis-associated AKI. Nevertheless in brain injured patients cellular mechanisms of subclinical AKI have not been fully elucidated.

We hypothesized that following TBI, tubular cells represent a target of the inflammatory reaction leading to a consequent cellular dysfunction. To test this hypothesis, we performed a prospective observational clinical study to identify severe TBI patients with subclinical AKI and increased inflammatory reaction. In the *in vitro* study, we exposed human kidney-derived tubular epithelial cells to plasma of TBI patients with evidence of subclinical AKI.

## Methods

### Clinical study

The Institutional Review Board (Comitato Etico Interaziendale AUO Città della Salute e della Scienza di Torino, Italy) approved this prospective observational study. If the patients were incompetent, they were included into the study and informed consent was delayed. The family was informed of the study (although not required). Written informed consent for using collected data was hence obtained from the patient (if competent) or from the family (in case of death or if the patient remained incompetent). All methods were performed in accordance with the relevant guidelines and regulations.

All patients with severe TBI consecutively admitted to the NeuroIntensive Care Unit at the Trauma Center of the “Azienda Ospedaliera Univerisitaria Città della Salute e della Scienza di Torino” were included as part of a European network for high resolution data collection after TBI, the Brain Monitoring with Information Technology^17^, during 18 months from September 2011 up to March 2013.

Inclusion criteria were age older than 18 years and severe traumatic brain injury (Glasgow Coma Score lower than 9). The severity of brain injury defined as GCS < 9 was confirmed after patient stabilization in ICU.

Exclusion criteria were GCS = 3, history of known chronic kidney disease (eGFR < 60 ml/min: Chronic Kidney Disease Epidemiology Collaboration (CKD-EPI) formula^18^) including diabetes; patients with extracranial injuries causing severe hemorrhagic shock and requiring transfusion on admission; immunosuppression, pregnancy, lack of consent.

#### Clinical management

Patients were managed according to the Brain Trauma Foundation Guidelines^19^. All patients were sedated, intubated and mechanically ventilated. Glasgow Coma Scale (GCS), Injury Severity Score (ISS) for the assessment of multiple injuries, Acute Physiology and Chronic Health Evaluation score (Apache II) to quantify the severity of illness and Marshall scale to classify head CT scan lesions were recorded on admission.

During the ICU stay, patients were continuously monitored to detect episodes of secondary insults (i.e. hypotension). Acute kidney injury (AKI) was assessed using the RIFLE criteria: serum creatinine (SCr) and 24 hrs urine output^20^, haemoglobin, white blood cell count.

#### Study protocol

*Blood samples* for analysis of Neutrophil Gelatinase-Associated Lipocalin (NGAL) and cytokine levels were collected at the time of inclusion (T0), at T24, T48 and T72 h. All plasma samples were centrifuged and then frozen at −80 °C until analyzed.

For the quantitative measurement of NGAL concentration (range from 60 to 1,300 ng/ml), the Triage® NGAL test was used, a fluorescence-based immunoassay used in conjunction with the Triage Meter (Biosite Inc., San Diego, CA), a fluorescence spectrometer.

The Bioplex assay for 27 cytokines was carried out in 96-well microplates using the Bio-Plex Pro Human Cytokine 27-plex Assay kit (Code M50-0KCAF0Y, Bio-Rad Laboratories version 6.1 with five-parametric curve fitting) at the Bioclarma - Research and Molecular Diagnostics, Torino, Italy. Plasma samples were diluted at 1:4 and run in duplicate.

Urine immunoelectrophoresis: Urine samples were collected at T48 for the evaluation of proteinuria; due to the emergency admission it was not possible to perform this analysis on fresh urine samples at T0 and on T48 were available in 10 patients. Determination of proteinuria was performed by the Hydragel 5 Proteinurie KIT (Sebia, Norcross, GA). Electrophoresis on neutral buffered SDS-agarose gels separated the urinary proteins by molecular weight and distinguished the proteins of tubular (<70 kDa: i.e alpha1-microglobulin and retinol binding protein (RBP)) and glomerular (>70 kDa) origin. The resulting electrophoretic patterns, stained with acid violet, were visually compared to molecular weight protein markers in the reference track to identify the individual proteins and thus classify proteinuria. Pooled samples for age- and gender-matched healthy volunteers served as controls.

### *In vitro* study

#### Isolation and culture of human proximal tubular epithelial primary cultures (PTEC)

PTEC were obtained from normal cortex fragments of surgically removed kidneys. Immortalized tubular cell line is generated by infection with a hybrid Adeno5/SV40 virus as previously described^21,22^.

Cell cultures were maintained in RPMI (Gibco BRL Life Technologies Inc., Gaithersburg, MD) containing 10% of fetal bovine serum (Gibco BRL Life Technologies Inc.) at 37 °C in humidified air with 5% CO_2_. PTEC were stimulated with 10% of patients’ plasma. Plasma from healthy volunteers were used as control. Optimal plasma percentage was determined on the basis of previous studies conducted in our laboratory^23^. In all the parts of the *in vitro* study PTEC monolayers were incubated with 10% TBI patients’ or healthy volunteers’ plasma.

#### Neutrophil adhesion assay

Neutrophils were isolated from healthy adult volunteer’s blood sample according to published methods^24^.

Whole blood added with EDTA was collected, layered over a density gelatin gradient and centrifuged. The neutrophil layer was aspirated and residual erythrocytes lysed. Cells were then washed, counted and re-suspended in buffer to obtain the desired concentration. Neutrophils were labeled with PKH26 red fluorescent cell linker kit (Sigma Chemical Co., Crailsheim, Germany). Confluent monolayers of PTEC in 48-well plates were previously incubated with 10% of TBI or healthy plasma for 8 hours. PKH26-labeled PMN were added to the monolayers and the plates were incubated at 37 °C with 5% CO_2_. After 30 minutes, non-adherent cells were removed and the monolayers were washed several times with 1X PBS. At this point, adhered PMN were counted using a fluorescence microscope.

#### Cell polarity assay

Cell polarity was assessed by Trans-Epithelial Electrical Resistance (TEER) and by expression of proteins typical of fully differentiated PTEC such as the endocytic receptor megalin^23^.

PTEC were plated on Transwells^®^ collagen-coated polycarbonate membranes (Corning Costar Corp., Cambridge, MA) and allowed to reach confluence before the addition of 10%of TBI patients’ plasma or control healthy plasma incubated for 12 h. An epithelial volt-ohm meter (EVOM, World Precision Instruments, Inc. Sarasota, FL. USA) was used to determine TEER values. All measurements were performed in triplicate for each well, normalized for the area of the membrane used in the experimental procedure and expressed as ohm/cm^2^.

#### Quantitative real-time (qRT-PCR) analysis for Megalin and NGAL

Total RNA was extracted using the RNeasy Mini Kit (Qiagen, Chatsworth, CA, USA).

RNA yield and quality was determined using a NanoDrop Spectrophotometer (NanoDrop Technologies, Wilmington, Delaware USA). Complementary cDNA was generated by reverse transcription of 2 μg of high quality total RNA using the High Capacity cDNA Reverse Transcription Kit (Applied Biosystems, Foster City, CA). The measurement of Megalin and NGAL mRNA levels was performed by SYBR green qRT-PCR analysis using the ABI Prism 7900 Sequence Detection System (Applied Biosystems, Foster City, CA) and SYBR FAST (Applied Biosystems, Foster City, CA). The following primers were used: Megalin forward 5′GCCGATGCATTTATCAAAAC3′, Megalin reverse 5′TCACATCCATCTTCATCTCC3′, NGAL forward 5′GGAAAAAGAAGTGTGACTACTG3′, NGAL reverse 5′GTAACTCTTAATGTTGCCCAG3′, GAPDH forward 5′ACAGTTGCCATGTAGACC3′, GAPDH reverse 5′TTTTTGGTTGAGCACAGG3′ (Sigma Aldrich, St. Louis, Missouri, USA). Relative quantification was performed using the standard curve method. The results for the target gene expression were normalized on GAPDH as the endogenous control, and the mean values of the vehicle were set as the calibrator.

#### Cell viability assay

Cellular viability was studied by using the XTT-based colorimetric method (Trevigen Inc., Gaithersburg, MD), a sensitive kit for the measurement of cell proliferation based upon the reduction of the tetrazolium salt, 2, 3-Bis(2-methoxy-4-nitro-5-sulfophenyl)-2H-tetrazolium-5-carboxanilide. The viability of the cells stimulated with TBI plasma was compared to that of the cells stimulated with healthy control plasma. Cells were seeded into 96-well plates with 150 μl of medium RPMI and incubated with 10% of TBI and control plasma for 24 hours. Then, XTT solution was added and the optical density was measured at 490 nm^25^.

#### Apoptosis assay

PTEC cells were exposed to 10% of TBI patients’ plasma for 48 hours. In all groups studied, apoptosis was assessed by terminal de-oxynucleotidyl transferase dUTP nick end labeling (TUNEL) assay and by the detection of fragmented DNA using the ApopTag® Plus *In Situ* Apoptosis Fluorescein Detection Kit (Millipore, Billerica, MA)^25^.

### Statistical analysis

Continuous data are presented as mean and standard deviation or median and range. Correlation between plasmatic NGAL and cytokines or proteinuria was performed by linear regression analysis. Data normally distributed were analyzed by Student’s t-test, and data not normally distributed were analyzed using a Mann-Whitney U or Kruskal-Wallis test. Differences were considered significant when the probability value was <0.05. Statistical analysis was performed using SPSS-statistical software, version 21.0 (SPSS Inc. Chicago, IL).

## Results

### Clinical study

**Figure 1:**
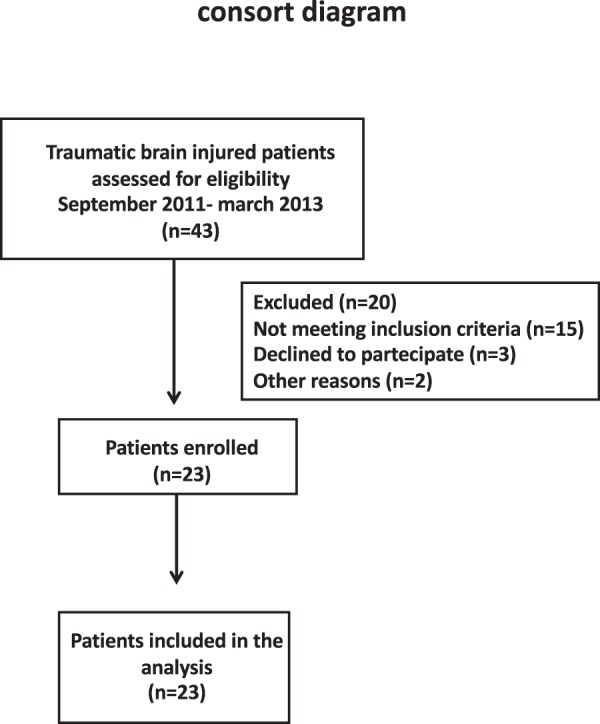
Patient enrollment flow diagram. Fourthy-three trauma patients were screened and 20 were excluded for the following reasons: 15 did not meet inclusion criteria for previous kidney disease (n = 5), for severe hemorrhagic shock (n = 10), in 3 was not possible to obtain consent and 2 for logistic problems.

Twenty-three adult severe TBI patients (19 males and 4 females) were enrolled in this study over a period of 30 months. Forty-three trauma patients were screened for eligibility (see Fig. 1: consort diagram). Demographic data are reported in Table 1. According to the ISS some of the patients had extracranial injuries but in none of them these injuries caused episodes of severe hypotension and none required transfusion. Therefore we excluded that acute kidney injury could be related to extracranial injuries. No patients developed AKI or were considered at risk according with RIFLE criteria: on T48 NGAL was elevated in 40%, RBP in all patients, α1-microglobulin in 10% and albumin in 90% of the patients compared to controls (Table 2).

**Table 1:** Demographic data of the patient population.

Patients variables (n = 23)	values
Age (yrs) mean (SD)	39 (19)
ISS mean (SD)	28 (11)
Apache II mean (SD)	15 (3)
GCS median (IQR)	5 (6; 3)
GCSm median (IQR)	2 (4; 1)
Marshall scale median (IQR)	4 (5; 3)

ISS injury severity score, Apache II Acute Physiology and Chronic Health Evaluation score, GCS Glasgow coma scale, GCSm Glasgow coma scale, motor score, TBI traumatic brain injury.

**Table 2:** Renal function and inflammatory mediators in patient population.

Parameters	Controls	T_0_	T_24_	T_48_	T_72_
SOFA		6 ± 2	8 ± 2	8 ± 2	8 ± 2
RIFLE		No risk	No risk	No risk	No risk
IL-6 (pg/ml)		146 (27–757)	116 (20–1000)	79 (18–1597)	74 (19–1382)
MCP-1 (pg/ml)		99 (5–2164)	234 (43–3320)	287 (43–3318)	210 (38–3278)
MIP1-β (pg/ml)		107 (25–772)	98 (38–1042)	125 (42–908)	134 (39–921)
NGAL (ng/ml)	<100		—	66 (17–337)	—
Serum creatinine (mg/dl)	0.60–1.30	0.79 ± 0.16	0.73 ± 0.10	0.70 ± 0.15	0.70 ± 0.13
Proteinuria (mg/g urine creatinine)	<100	—	—	197 (40–793)	—
Retinol Binding Protein (mg/g urine creatinine)	<1	—	—	5.1 (1–27)	—
α1-microglobulin (mg/g urine creatinine)	<14	—	—	55 (3.6–212)	—
Urinary albumin (mg/g urine creatinine)	<20	—	—	38.4 (25–76)	—

Data are presented as mean ± standard deviation (SD) or median and range.

SOFA: Sequential Organ Failure Assessment; IL-6: interleukin 6; MCP1: monocyte chemoattractant protein 1; MIP1-**β**: macrophage inflammatory protein; NGAL: Neutrophil Gelatinase-Associated Lipocalin.

**Figure 2:**
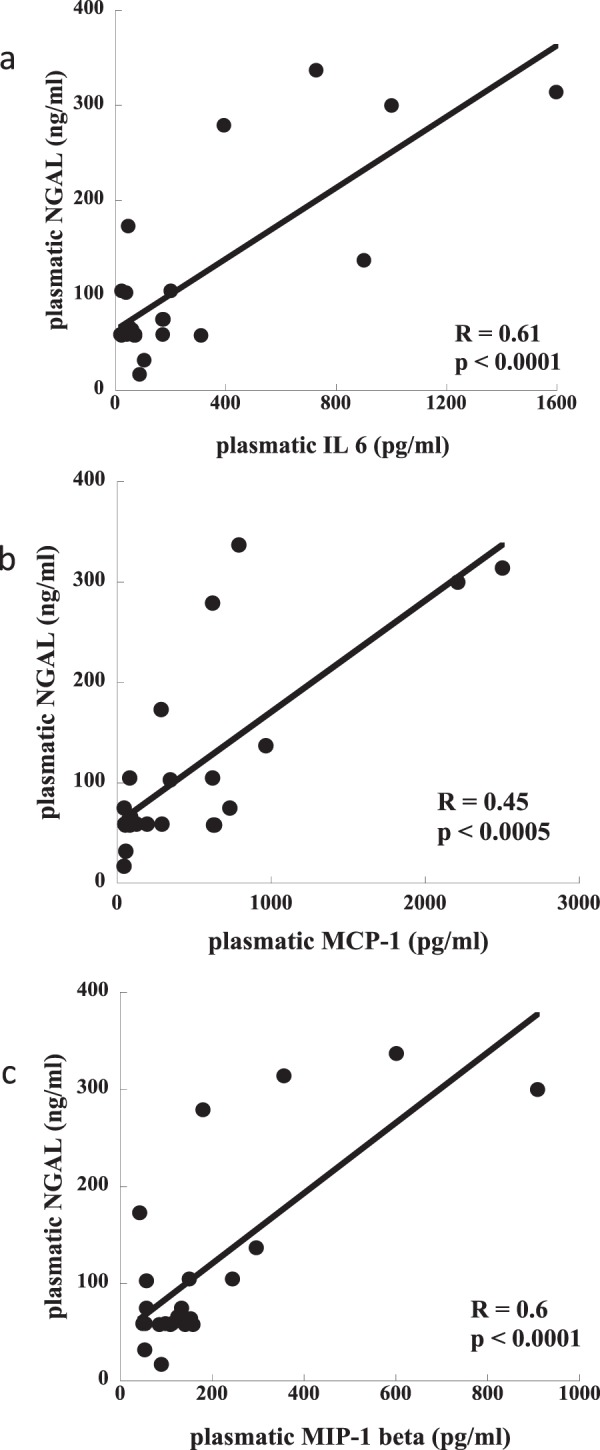
Correlation between concentration of plasmatic NGAL and inflammatory mediators. (n = 23): panel a) NGAL vs IL-6 (R^2^ = 0.53, p < 0.0001); panel b) NGAL vs MCP-1 (R^2^ = 0.45, p < 0.0001); panel c) NGAL vs MIP-1β (R^2^ = 0.6, p < 0.0001).

**Figure 3:**
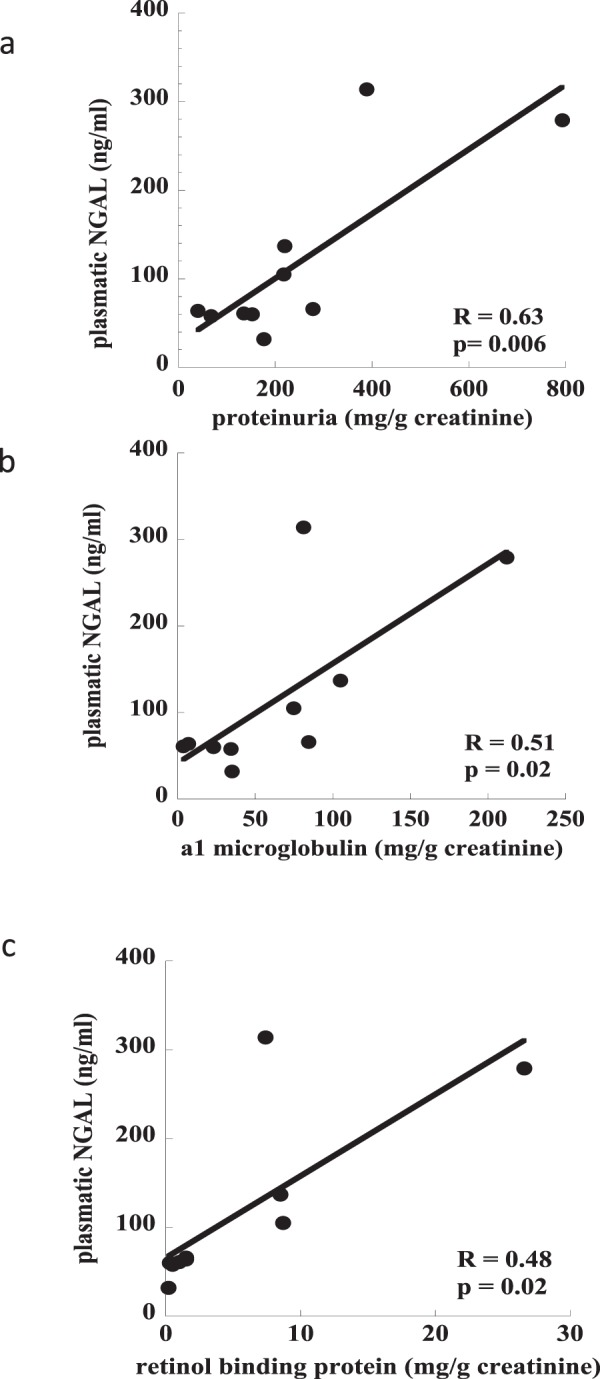
Correlation between concentration of plasmatic NGAL and markers of tubular damage (n = 10): panel a) NGAL vs total proteinuria (R^2^ = 0.58, p = 0.006); panel b) NGAL vs α1-microglobulin (R^2^ = 0.46, p = 0.019); panel c) NGAL vs Retinol Binding Protein (R^2^ = 0.45, p = 0.02).

Among the measured cytokines (data partially published in a previous study^26^) those with the strongest correlation with markers of tubular damage were selected for the present analysis. Plasmatic NGAL and inflammatory mediator levels collected on T48 were significantly correlated: NGAL vs IL-6 (R^2^ = 0.61, p < 0.0001), NGAL vs MCP-1 (R^2^ = 0.45, p < 0.0005) and NGAL vs MIP-1β (R^2^ = 0.6, p < 0.0001; Fig. 2a–c). Plasma NGAL levels were also significantly correlated with total proteinuria values and markers of tubular damage: proteinuria (R = 0.63, p = 0.006), α1-microglobulin (R = 0.51, p = 0.02) and RBP (R = 0.48, p = 0.02) respectively (Fig. 3a–c; n = 10).

### *In vitro* study on proximal tubular epithelial cells (PTEC)

**Figure 4:**
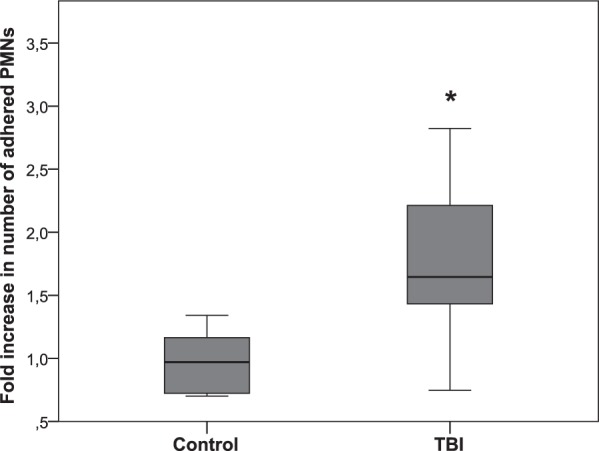
Adhesion of neutrophils on PTEC monolayers incubated with 10% of TBI patients’ plasma (n = 23) compared to control group (n = 7) for 8 hours. Comparison between groups was made by unpaired t test (*p = 0.001; and data are presented as box plot with median and IQ1-IQ3).

PMN adhesion significantly increased after stimulation of PTEC with TBI plasma compared to control (Fig. 4, p = 0.001).

**Figure 5:**
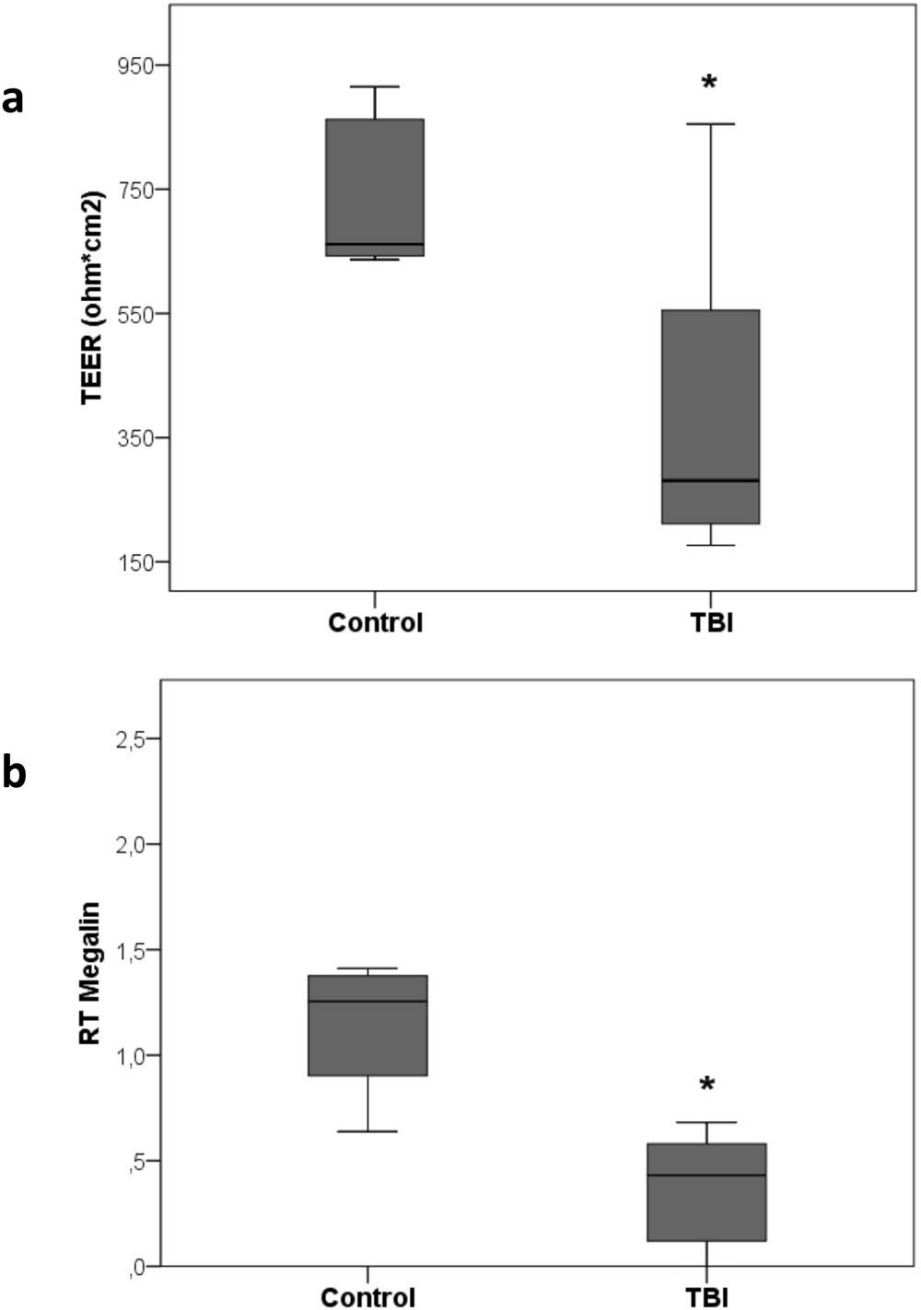
Tubular dysfunction: panel a) alteration of PTEC polarity induced by TBI plasma incubation for 12 hrs. Comparison between TBI and control groups was made by test U Mann-Whitney, unpaired (*p = 0.003). Panel b) qRT-PCR analysis of Megalin transcript levels in PTEC. Results were normalized to GAPDH expression. Comparison between TBI (n = 23) and control (n = 4) groups was made by test U Mann-Whitney, unpaired (*p = 0.008; data are presented as box plot with median and IQ1-IQ3).

PTEC stimulated for 12 hours with TBI plasma showed significantly reduced levels of TEER compared to control plasma (Fig. 5a, p = 0.003).

Megalin mRNA was significantly down-regulated after incubation with TBI compared to control plasma (Fig. 5b, p = 0.008).

**Figure 6:**
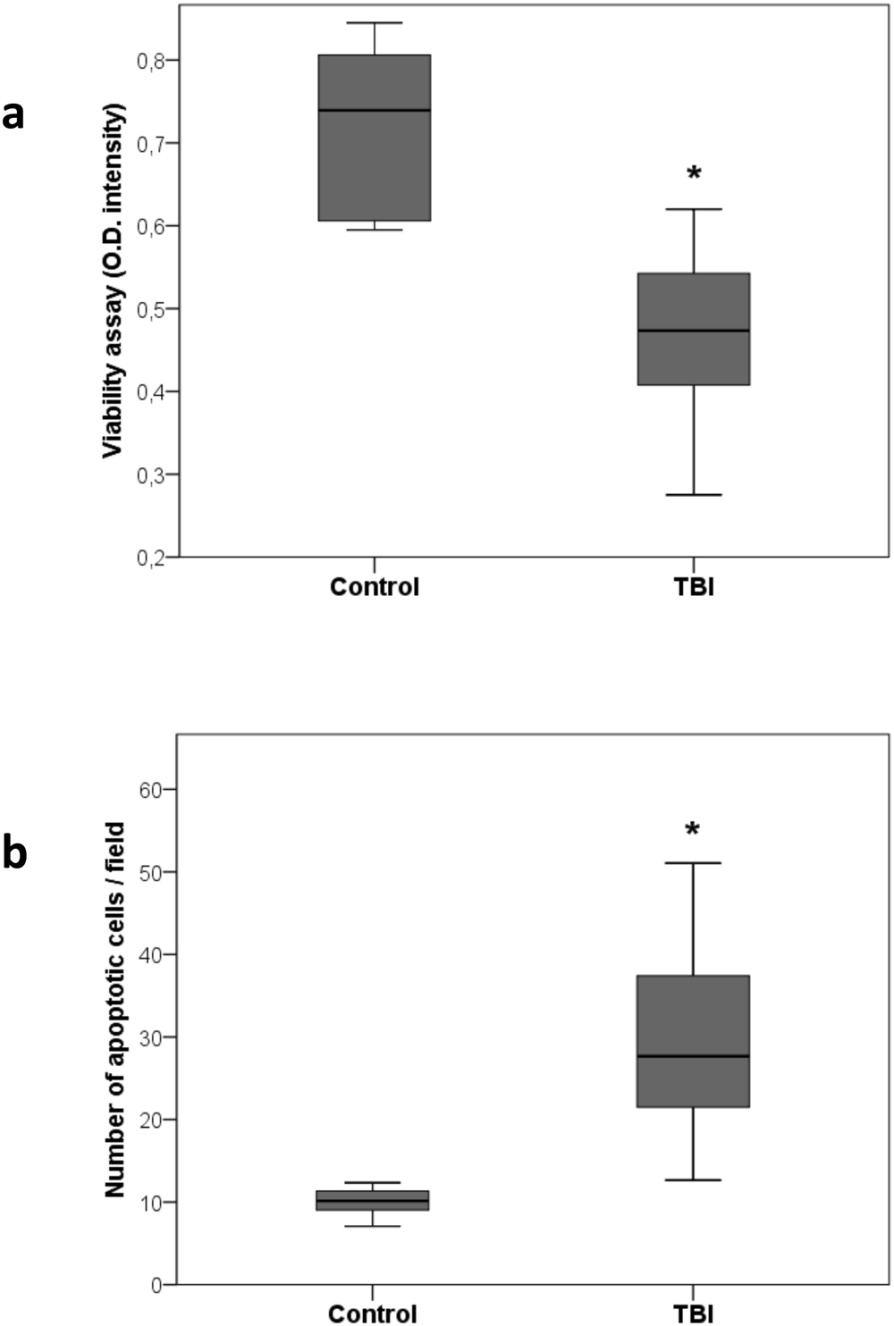
Tubular cells damage: Effect on PTEC viability (panel a) and apoptosis (panel b) after incubation with TBI plasma for 24 and 48 hrs respectively. Comparison between TBI (n = 23) and control (n = 6) groups was made by unpaired t test (*p = 0.0001; data are presented as box plot with median and IQ1-IQ3).

After 24 hours of incubation, TBI plasma induced a significant cytotoxic effect on cultured PTEC compared to control plasma as detected by the XTT-based assay (Fig. 6a, p = 0.0001). The cytotoxic effect exerted by TBI plasma on PTEC was ascribed to the triggering of the apoptotic pathway as confirmed by evaluation of DNA fragmentation by TUNEL assay. Indeed, exposure of PTEC for 48 hours to TBI plasma induced a significant increase of apoptosis compared to control plasma (Fig. 6b, p = 0.0001).

## Discussion

This study demonstrates for the first time that patients with TBI exhibited both increased urinary levels of low molecular weight proteins and plasmatic NGAL even though serum creatinine levels were normal. These markers were positively correlated with inflammatory mediators, suggesting the presence of tubular dysfunction related to the inflammatory reaction. In the *in vitro* study, plasma of TBI patients induced functional alterations and apoptosis of human tubular epithelial cells. Some patients had extracranial injuries, but they were never responsible for severe hypotension requiring transfusion and consequently acute kidney injury.

These results suggest that the presence of acute cerebral damage makes the kidney vulnerable for tubular dysfunction (the so called “subclinical AKI”) and that tubular injury is probably associated with the severity of the systemic inflammatory response. Indeed plasma NGAL and low molecular weight proteinuria were significantly increased compared to controls even if biomarkers currently used for diagnosis and risk of AKI (i.e. serum creatinine and estimated glomerular filtration rate (eGFR))^26–31^ were not altered. NGAL is released by neutrophils, hepatocytes and epithelial cells during inflammation and/or injury. This protein has been proposed as biomarker of AKI being up-regulated in the distal tubules early after ischemic and toxic injury^32^.

The increased plasmatic NGAL concentrations observed in the present study could be related to the inflammatory reaction occurring after TBI as well as to the development of AKI. The parallel increase of NGAL levels and proteinuria suggested the presence of a subclinical form of AKI. Indeed α-1microglobulin and RBP are low molecular weight proteins able to pass across the glomerular membrane into the Bowman’s capsule and then re-adsorbed by the proximal tubule through a mechanism involving the endocytic receptor megalin^33^. The presence of low molecular weight proteinuria in TBI patients confirmed the putative presence of an ongoing subclinical AKI associated with tubular dysfunction. In future larger studies including patients with increased serum creatinine it would be interesting to investigate if there is a threshold level of NGAL corresponding to increased creatinine level.

The damage of tubular epithelial cells represents a hallmark of both ischemic and nephrotoxic AKI. Although several clinical studies reported an increased incidence of AKI in TBI patients, the mechanisms of renal damage have not been fully elucidated. Indeed, AKI develops even without severe episodes of hypoperfusion or exposure to nephrotoxins and tubular injury may be correlated to TBI-associated systemic inflammation and secondary organ damage.

On this basis, the mechanisms of TBI-associated renal dysfunction may be similar to those observed in experimental and clinical studies in patients with severe sepsis where AKI may be mainly ascribed to microvascular derangement as well as to functional alterations of tubular epithelial cells including loss of solute carrier expression, bioenergetic alterations due to an impairment of mitochondrial function and, ultimately, apoptosis^34^. We previously found that plasma from patients with severe sepsis contains factors able to induce functional alterations of tubular and glomerular epithelial cells^23^. Part of inflammatory mediator data has been previously published from our group showing a significant correlation with secondary injury^26^. In the present study, we demonstrated that plasma from TBI patients vehicles molecules involved in the development of tubular dysfunction^13,35^. Moreover, it has been shown that IL-6 mediates a cytokine-dependent cell-mediated immune response that may exacerbate renal injury playing a critical role in the inflammatory response associated with AKI^16^. MCP-1 and MIP-1β are chemokines produced by leukocytes and endothelial cells during tissue injury: these molecules are involved in neutrophil infiltration attracting inflammatory cells from circulation into injured tissues^36^. Viedt and coworkers demonstrated that MCP-1 activates human tubular epithelial cells leading to a time- and dose-dependent increase of IL-6 and ICAM-1 secretion. Moreover, apart from its chemoattractant activity, MCP-1 may further increase the inflammatory response by inducing cytokine release and adhesion molecule expression in tubular epithelial cells^37^.

In the *in vitro* study, PTEC were challenged with plasma of TBI patients in order to confirm the hypothesis of subclinical AKI generated by clinical observations. Plasma of TBI patients with subclinical AKI induced in PTEC neutrophil adhesion, altered permeability, neutrophil adhesion, reduced viability, enhanced apoptosis and increased expression of inflammatory molecules.

First, we found an increase in neutrophil adhesion to tubular epithelial cells. Adhesion is the initial stage of neutrophil trans-epithelial migration. Binding of neutrophils to the apical epithelium is known to be mediated by specific adhesion molecules such as ICAM1, expressed predominantly on the apical surface of epithelial cells^38–40^ and up-regulated in response to inflammatory stimuli^38,39,41–43^. It has been shown that neutrophils are able to injury the epithelium through several mechanisms, such as regulating disassembly of tight junctions, mechanical force, and degradative effects of soluble mediators including elastases, matrix metalloproteinases, defensins and oxidants^44^. Different studies provided evidence that neutrophil transmigration through an epithelial monolayer is sufficient by itself to trigger apoptotic cell death^44,45^. However, tubular cell apoptosis in the course of systemic inflammation can be ascribed to an alteration of mitochondrial biogenesis and function as well as to the presence of pro-apoptotic circulating mediators able to interact with specific receptors located on tubular cells^46^. In this study, we found that after long term incubation with TBI plasma, tubular cells underwent apoptosis. The persistence of apoptotic tubular injury may lead to the possible lack of tissue regeneration leading to a maladaptive repair: on this basis, innflammation may have a relevant role in the extension phase of AKI and progression toward fibrosis and consequent development of chronic kidney disease^46^. Infiltrating inflammatory cells may release an additional load of harmful mediators able to perpetuate tubular injury.

Polarity is a distinctive feature of epithelial cells mandatory to maintain two separate compartments with precise electrolyte concentrations. The mislocalization of specific solute carriers on tubular cell surface may lead to an increased influx of sodium and chloride in the distal tubule, thus leading to the triggering of tubulo-glomerular feedback with a consequent reduction of glomerular filtration rate. Moreover, the altered expression of other molecules usually located on the apical surface of PTEC may be responsible for typical alterations such as proteinuria observed in patients with sepsis and confirmed in our cohort of TBI patients.

Megalin is a multiligand, endocytic receptor expressed in kidney proximal tubule luminal membranes and apical endocytic compartment that is responsible for normal tubular reabsorption of several filtered proteins, mediating the recovery of essential substances that would be otherwise lost in the urine^47^. As we previously observed in septic patients with AKI, we found a significative decrease of megalin mRNA levels in PTECs incubated with TBI plasma.

The presence of tubular cell dysfunction and apoptosis demonstrated in our *in vitro* study generates the hypothesis that subclinical AKI is underestimated in TBI patients using conventional biomarkers of renal function. This vulnerability of the kidney may evolve to clinical damage potentially leading to worse outcome.

Furthermore, almost 10% of severe brain damaged patients may evolve to brain death and then may become potential organ donors^48,49^. The presence of an unidentified and persistent tubular injury may therefore be clinically relevant in the setting of kidney transplantation. The tubular dysfunction associated with TBI may enhance kidney transplant damage due to ischemia-reperfusion injury and drug toxicity contributing to increase the incidence and duration of delayed graft function. On this basis, the use of functional and structural biomarkers of tubular damage in TBI patients should be encouraged.

### Limitations of The Study

The main limitations of the present study are related to the small number of patients and to the co-presence of extracranial injuries.

- In our patient population brain trauma was associated in more than 50% of the cases with extracranial injuries, but we did not include patients with severe hemorrhagic shock requiring transfusion to avoid that the severity of multiple trauma was the leading process starting inflammation and that a severe and prolonged hypothension was the main cause for tubular injury. Indeed it has been shown that in severe traumatic brain injured patients an inflammatory reaction is correlated both to the severity of primary injury and to the secondary insults occurring during the ICU stay^10,26^. We therefore tested the hypothesis that the inflammatory reaction triggered by the brain trauma could contribute to the development of tubular damage.
- We performed an observational study in a small number of patients, indeed only a low percentage developed AKI according to KDIGO criteria. Further studies including large cohort of severe brain injured patients are required to further elucidate the mechanism of tubular injury following traumatic brain injury.

## Conclusions

To the best of our knowledge, we observed for the first time the consequence of TBI on renal function correlated to the inflammatory process associated with brain injury. TBI triggers a complex cascade of molecular and cellular events associated with systemic inflammation and secondary distal organ damage. Circulating pro-inflammatory cytokines and chemokines such as IL-6, MCP-1 and MIP-1ß are potentially responsible for a hidden form of AKI that may progress toward chronic kidney disease without manifest signs. These considerations are suggested both by the presence of increased levels of NGAL and low molecular weight proteinuria in the enrolled patients and by the evidence of tubular cell dysfunction and apoptosis induced in PTEC incubated with TBI plasma.
